# The Charlie Gard case: British and American approaches to court resolution of disputes over medical decisions

**DOI:** 10.1038/jp.2017.138

**Published:** 2017-10-19

**Authors:** J J Paris, J Ahluwalia, B M Cummings, M P Moreland, D J Wilkinson

**Affiliations:** 1 Department of Bioethics, Boston College, Chestnut Hill, MA, USA; 2 Campion Hall, Oxford University, Oxford, UK; 3 Addenbrookes Hospital, Cambridge, UK; 4 Department of Pediatrics, Massachusetts General Hospital, Boston, MA, USA; 5 Villanova University School of Law, Villanova, PA, USA; 6 John Radcliffe Hospital, Oxford, UK; 7 Oxford Uehiro Center for Practical Ethics, University of Oxford, Oxford, UK

For those whose bodies are riddled with disease, [Aclepius] did not attempt to prescribe a regimen in order to make their life a prolonged misery. Medicine isn’t intended for such people... even if they are richer than Midas. Plato, *The Republic*, Bk III.

## Introduction

On 8 June 2017, the Supreme Court in the United Kingdom rejected a legal appeal in the high-profile case of Charlie Gard, a British infant with a severe genetic disorder whose parents had disagreed with medical professionals and were requesting treatment that the doctors believed was futile.^1^ The case was the latest in a series of UK legal cases where courts have authorized withdrawal of treatment against the wishes of parents. In such disputes, British judges have, with rare exception, sided with health professionals. In contrast, in North America when disputes have reached the court, the courts have invariably sided in favor of life-sustaining medical treatment requested by a loving family.

In the United Kingdom and United States such disagreements are generally resolved in discussions between the family and the doctors with, if necessary, the assistance of a hospital ethics committee.^2^ If the conflict proves intractable, one of the parties might seek a court order in support of its position.

## The case of Charlie Gard

Charlie Gard was a 9-month-old infant who had been in an intensive care unit at London’s Great Ormond Street Hospital (GOSH) for more than 6 months.^3, 4^ He was being treated for a rare genetic condition called encephalomyopathic mitochondrial DNA depletion syndrome (MDDS). His physicians and a number of external consultants were unanimous that there was no known treatment for his form (RRM2B) of the disorder. Charlie’s treating physicians believed that he could probably experience pain and that there was nothing further that medicine could do to alter Charlie’s condition or benefit him. His physicians proposed withdrawing mechanical ventilation and allowing him ‘to die with dignity.’

Charlie’s parents did not accept the British doctors’ assessment. They desperately wanted to try an experimental treatment (nucleoside therapy) that had been proposed by a doctor in the United States identified by the court as ‘Dr I.’ To finance the treatment the parents raised £1.3 million. The disagreement between the parents and the physicians in this case was not about cost, it was whether or not the experimental therapy ought to be tried on Charlie. The parents wanted it done. The physicians were opposed. They believed that continued intensive care and the proposed nucleoside treatment were ‘futile.’

A British Court was petitioned to approve an order ‘that it is lawful, and in Charlie’s best interests, for artificial ventilation to be withdrawn... and for his treating clinicians to provide him with palliative care only.’ The request was a direct challenge to the parent’s hopes.

The case was assigned to Mr Justice Francis of the Family Division of the High Court. The judge made clear from the outset that ‘This case is not about money.’ As he put it ‘If anyone were to support that Charlie would have nucleoside treatment but for the cost [to the NHS], they would be completely wrong.’ Cost, though, he acknowledged, is a factor for medical treatment in the United States. In his words, ‘The US doctor made it clear that were Charlie in the United States, he would treat him if the parents so desired *and could pay for it*.’ (italics added).

In a detailed opinion, Mr Justice Nicholas Francis wrote ‘No one in the world has ever treated this form of MDDS with nucleoside therapy.’ That therapy has been used with a different mutation (TK2) with some limited success (a 4% increase in life expectancy), but as the judge noted, ‘There is no evidence that nucleoside therapy can cross the blood/brain barrier which it must do to treat RRM2B.’ In testimony by telephone ‘Dr I’ acknowledged the therapy had never been tested, even in animal studies, for the mutation afflicting Charlie, and agreed the damage to Charlie’s brain was largely ‘irreversible’^4^.

## Requests for UNPROVEN treatment: US experience

A similar diagnosis of irreversible brain damage was made by physicians in the near-drowning of a 2-year-old Florida boy.^5^ The father sought an experimental therapy—hyperbaric oxygen therapy. In spite of the negative findings of a contemporaneous randomized multicenter trial published in *The Lancet*^6^ on the impact of hyperbaric oxygen therapy on anoxic brain damage, the father, desperate to try anything that might increase his son's chance of neurological recovery, contacted a local for-profit hyperbaric oxygen therapy clinic that treats children with cerebral palsy and brain injuries. When the pediatric intensivists treating the Florida boy declined to provide the proposed experimental therapy, the father took his case to the media and then to court.

The circuit judge in the Florida case, after finding that *the parents were willing to pay for the treatment and waive any liability*, issued an order directing the hospital to install a hyperbaric oxygen chamber in its facility. The child was discharged home after 40-h-long sessions of hyperbaric oxygen therapy without any change reported in his neurological status.^7^ What the Florida case demonstrates—and is seen in such well-documented US cases as Baby K^8, 9^ and Jahi McMath^10,11^—is that American courts are unwilling to order the cessation of life-sustaining treatment over the protests of a caring family. This proved true even in such extreme cases as *Baby K*, in which the infant was born without a brain, and in *Jahi McMath*, in which a court had ruled the child was ‘legally dead.’

## Evolving approach to ethical questions

Cases of family–physician conflict over treatment decisions present multiple ethical issues. Among the questions are: Who decides? On what standards? Are those standards different when experimental procedures are utilized?

Mr Justice Francis began his opinion in the *Gard* case by noting how the court became involved in what appears to be a rather common question in the care of a seriously ill patient: what medical treatments should be utilized? Thousands of such decisions are made daily in hospitals without court involvement. In much of the world they are made, as they have been since the time of Hippocrates, exclusively by the physician.^12^ (The Hippocratic Corpus advises, ‘Tell the patient nothing of his present of future condition.’ The rationale at the time was the view that ‘such information might upset, or still worse, harm the patient.’ Or it might frighten the patient into refusing a procedure that could be of significant benefit to the patient.)

That paternalistic approach has yielded in the United States to the recognition that the dignity of each human being provides the individual a ‘right of privacy,’ that is, ‘the right to be left alone.’^13^ Also accorded judicial protection is the right of a patient to be free of unwanted touching by a physician—even if the physician was attempting to benefit the patient.^14^ The right of ‘autonomy’ (or ‘self-determination’) finds its fullest expression in John Stuart Mill’s declaration, ‘Over his mind and his body every individual is sovereign.’^15^ A corollary of that position, found in Justice Benjamin Cardozo’s opinion in the 1914 *Schloendorff* case, is ‘Every human being of adult years and sound mind has the right to determine what shall be done with his own body.’^16, 17^ Cardozo continued ‘And a surgeon who performs an operation without his patient’s consent commits an assault for which he is liable in damage.’

The combination of the doctrine of privacy and the requirement of consent allows the competent patient to decline unwanted medical interventions. That ‘right’ was extended to the non-competent in a series of legal cases in the United States beginning with the New Jersey Supreme Court 1976 opinion *In re Quinlan*,^18^ and culminating in the 1990 US Supreme Court’s *Cruzan* opinion that recognized the right—subject to a particular state’s evidentiary standards—of an incompetent patient to decline any and all unwanted medical interventions.^19^ That negative moral right morphed into the claim that a patient’s right of self-determination in medical cases applies not only to the refusal of unwanted medical intervention, but to the right to demand and to receive whatever medical intervention was desired.^20, 21^

## Jurisdiction in the United Kingdom

One might inquire, as did Mr Justice Francis, ‘Why should the parents not be the ones to decide?’ Francis explained that ‘while a child’s parents have the power to give consent for their child to undergo treatment, in the United Kingdom overriding control is vested in the court exercising its independent and objective judgment in the child’s best interests.’ This principle, he reminded the public, has been enunciated over the years in many cases. He specifically referred to the ruling of Lord Justice Ward in the well-known conjoined twins case, *In re A (Children).*^22, 23^ He also referenced *Wyatt v. Portsmouth NHS Trust*,^24^ in which the Court of Appeal set out ‘intellectual mindstones’ to guide judges on how to resolve such disputes and *An NHS Trust v. MB*,^25^ in which Mr Justice James Holman provided practical directives on how a judge should proceed once such disputes reached the courts.

Holman insisted that the judge is not to decide what he himself would do in such a situation, nor whether the respective decisions of the parents or those of the physician are ‘reasonable.’ The focus is exclusively on ‘the best interests of the patient.’ He also stressed that the views of the parents are not dispositive but simply their personal opinion. In his forceful phrasing, ‘Their own wishes, however understandable in human terms, are wholly irrelevant to consideration of the objective ‘best interests of the child.’ Finally Francis in the *Gard* case cites Lord Donaldson’s ruling in the 1991 case of *Re J* that while there is a strong presumption in favor of prolonging life ‘in the end there will be cases in which the answer must be that it is not in the interests of the child to subject it to treatment which will cause it increased suffering and produce no commensurate benefit.’^26^

## Precedent in the United Kingdom

After a thorough review of legal precedent, including the ruling of the United Kingdom’s Supreme Court in *Airedale NHS Trust v. James*^27^ and the ruling in the House of Lords in *Airedale NHS Trust v. Bland*,^28^ Mr Justice Francis highlighted the directives from the United Kingdom’s Supreme Court in the *James* case that ‘decision makers must look at [the patient’s] welfare in the widest sense, not just medical, but social and psychological, they must consider the nature of the medical treatment in questions, what it involves and its prospects for success; they must consider what the outcomes of that treatment for the patient is likely to be; and they must try and put themselves in the place of the individual patient.’

Here the British courts, as Christopher Stone notes in a commentary on legal precedent in the United Kingdom, ‘build on, modify or ‘fine tune’’ the common law to accommodate for small but significant variations in the material facts of successive cases. In doing so, both the clarity and precedent of the law adjusts to new situations while providing consistency and guidance for future conflicts.^29^ For British courts, the precedent on family–physician conflict over treatment decisions generally tends to support the decision of the physician.

American courts, in contrast, almost always favor the ‘autonomy’ claims of the patient or family. Such an emphasis shifts the court’s focus from the ‘best interests’ of the incompetent patient to the putative claims of patient (or family) to ‘autonomy’ to determine what medical treatment the now non-competent patient would, if competent, choose. Vast, indeed, is the area for speculation or the opportunity for flights of fantasy by a process untethered by a concern for the best interests of the patient.

## Court system in the United States

The United States has no centralized decision making body to resolve family–physician conflicts over medical treatment. Their guidance is the domain of the legislature and courts of 50 various states, each of which may set its own standards. The wide range of views in the United States include those of the Massachusetts Supreme Judicial Court in *In re Saikewicz* that decisions on the withdrawal of life-sustaining medical treatment are of such moment they cannot be entrusted to families or doctors.^30^ Rather, the Supreme Judicial Court ruled such decisions are ‘our responsibility and that of the lower courts.’ Then in a criticism of the New Jersey Supreme Court’s ruling in the famous Karen Ann Quinlan case^31^—which left the decision on whether to remove the life-sustaining ventilator to Karen Ann’s parents, subject to confirmation by an ethics committee—the Supreme Judicial Court ruled ‘such decisions are not to be entrusted to any other group proposing to represent the morality and conscience of our society, no matter how highly motivated or impressively constituted.’

The New York Court of Appeals—that state’s court of final jurisdiction—went even further in an opinion in the *In re Storar*, a case concerning the medical treatment of a never competent patient in a state mental institution who, at age 80 years, was diagnosed with bladder cancer.^32^ Owing to the cancer, John Storar lost a unit of blood every 8–15 days. His mother, who had visited him every day since his admission to the state facility in 1942, asked the physicians to cease the pain-causing blood transfusions. A trial court judge agreed. The New York Court of Appeals reversed that judgment. In doing so, it characterized as ‘judicial fantasy’ the Supreme Judicial Court’s putative determination of what the never competent patient would have wanted. New York’s highest court ruled that a life-sustaining medical intervention could not be removed from anyone absent ‘clear and convincing’ evidence—from the patient when competent—that such was his will.

## Should the courts decide?

Pining for courts to resolve intractable disputes has been and continues to be an American fantasy. As early as 1835 in *Democracy in America*^33^ Alexis De Tocqueville observed what he labeled ‘the strange propensity of Americans to translate their moral dilemmas into legal problems,’ as if judges, unlike mere mortals, could transcend personal bias and politics in resolving difficult and trying moral issues. That propensity persists to this day. Medicine is not immune to that tendency. Robert Truog, the director of the clinical ethics program at Harvard Medical School in an audio link to his 2007 article in *The New England Journal of Medicine* on the resolution of family–physician conflicts on treatment decision, insists that the American tradition of ‘due process’ demands resolution by a ‘jury of one’s peers,’ that is, a court proceeding.^34^

Courts are, by nature adversarial, cumbersome and costly. They lack sensitivity to clinical situations.^35^ Furthermore, they are not structured to resolve medical disputes in a timely fashion. Nor, in adjudicating a particular case in controversy, does a trial judge have the time, staff or resources to explore complex and troubling issues in the depth required to establish sound public policy.

## The best interests of the child

After years of shifting standards on medical treatments, there is now a strong consensus in the medical and ethical literature in the United States that it is the best interests of the patient—not the desires of the family or the personal predilections of the physician—which ought to prevail.^36^ That standard does not rest on autonomy or an attempt to determine what the patient would have wanted, but solely on a concern for the patient's welfare. Such protection is particularly important with regard to infants and children because with it they are now seen not merely as the pawns of parents, but as patients in their own right.^37^ The implication is that although parents may continue to be involved in decision making for their children, they do not have an absolute right to refuse—or to require—medical treatment for their child. It is the child's best interests, and those alone, that are to be the focus and goal of medical treatment decisions made on behalf of children.

Translated into practice that standard means if the burden on the infant is overwhelming or the prospects are extremely bleak, as is true in the cases presented in this paper, there is no obligation to use intensive care.^38^

## Request for Experimental Treatment

The issue in the *Gard* case, however, is not that of parents wanting to stop treatment, but of parents’ desire to try an experimental therapy that has the potential for additional suffering to the child. Justice Francis found that the proposed treatment itself would not cause any harm (although there was evidence that it could have unknown and unpredictable effects) - rather, the harm was continued ventilation in the absence of any prospect of meaningful improvement in his condition. The harm aspect of the issue is the easier to resolve. The physician’s primary commitment to the patient, captured in the Hippocratic dictum, ‘First do no harm,’ precludes putting the child at substantial risk for research purposes.^39^ Although generally the risk–benefit assessment belongs to the patient or for children to the parents, where children are involved the physician as well as the state has an independent fiduciary duty of protecting a vulnerable child against even the well-meaning, but perhaps ill-informed or misguided, directives of the parents.^40^ That is true of therapeutic treatments provided to help the patient. The commitment not to put a child at ‘more than minimum risk’ is intensified when the proposed procedure involves an experimental therapy.^41^

Francis Moore warned physicians that before undertaking an untested procedure on a patient, ‘There must be a rationale on which the desperately ill patient may be offered not merely pain, suffering and cost, but also a true hope of prolonged survival [without devastating sequelae].’^42^ The untested experimental therapy proposed in the *Gard* case does not meet that criterion.

When the physician ventures beyond the proven to explore and test a new hypothesis or theory, the physician leaves the area of therapeutic medicine and enters, at best, into non-validated practice or investigational research. Informed consent alone, as the death of Jesse Gelsinger demonstrated, is not a sufficient basis on which to embark on such a venture.^43, 44^ As Moore has noted, there must be a well worked-out theoretical basis for the proposed intervention, careful laboratory studies on animals and extensive ‘field experience’ by the researcher before the physician submits a patient to such a process. And, as the Nuremberg Code highlights, even then, the physician has a heightened obligation to be aware of and prevent potential harm to the patient.^45^

These are a reminder that in medicine the indication for the use of the latest technology is not merely its availability, but its benefit to the patient. Absent such a benefit, even the most sophisticated technology or drug is as useless to a patient as the elixirs widely peddled by snake oil salesmen before there were regulations on the safety and efficacy of drugs.^46^

## Differences between British and American approaches

Owing to differences in their health-care systems, inclination to litigiousness, and a greater sense of a common community, fewer disputes are brought to the courts in the United Kingdom than in the United States. When cases do make their way to court, the British courts view issues not from the perspective of patient or family ‘autonomy,’ but the ‘best interests’ of the patient.

Another major difference in the systems is payment for litigation. The ‘contingency fee’ approach used in civil cases in the United States allows individuals who believe they were unjustifiably injured in the medical context to seek legal assistance for one-third of any future payment for the claim. There are no immediate ‘out of pocket’ costs for initiating a law suit. Seeking a legal remedy for a perceived injury in the United Kingdom is a more expensive proposition. Significant ‘out of pocket’ costs are a disincentive to seeking a court remedy for a complaint though means-tested legal aid is available to families in these cases.

Finally, the unitary court structure in the United Kingdom promotes a greater consistency in judicial rulings than is found in the United States. In the United Kingdom once a higher court promulgates a way to proceed and established a standard to be followed, lower courts tend to conform. In the United States, the multiplicity of jurisdictions established under the Constitution precludes such a unified legal system.

## Conclusion

The UK Court system—although not perfect and subject to the problems that plague all attempts at judicial resolution on dispute over medical treatment—in its timeliness, consistency and attention to what matters most (the interests of the child) provides a model of how the judicial system should respond to family–physician conflicts over treatments for seriously ill children.

Given Mr Justice Francis’ careful analysis in the *Gard* case, the thoroughness of his research and his faithfulness to the guidelines provided by higher courts on resolving family–physician conflicts on treatment decisions, it is no surprise that his ruling in the *Gard* case—heartbreaking as it was —was unanimously upheld by the Court of Appeal and the UK Supreme Court. On 27 June 2017, the European court of human rights rejected the parents’ appeal, closing the final avenue of appeal.^47^
